# MoMA-LigPath: a web server to simulate protein–ligand unbinding

**DOI:** 10.1093/nar/gkt380

**Published:** 2013-05-11

**Authors:** Didier Devaurs, Léa Bouard, Marc Vaisset, Christophe Zanon, Ibrahim Al-Bluwi, Romain Iehl, Thierry Siméon, Juan Cortés

**Affiliations:** ^1^CNRS, LAAS, 7 av du colonel Roche, F-31400 Toulouse, France and ^2^Univ de Toulouse, LAAS, F-31400 Toulouse, France

## Abstract

Protein–ligand interactions taking place far away from the active site, during ligand binding or release, may determine molecular specificity and activity. However, obtaining information about these interactions with experimental or computational methods remains difficult. The computational tool presented in this article, MoMA-LigPath, is based on a mechanistic representation of the molecular system, considering partial flexibility, and on the application of a robotics-inspired algorithm to explore the conformational space. Such a purely geometric approach, together with the efficiency of the exploration algorithm, enables the simulation of ligand unbinding within short computing time. Ligand unbinding pathways generated by MoMA-LigPath are a first approximation that can provide useful information about protein–ligand interactions. When needed, this approximation can be subsequently refined and analyzed using state-of-the-art energy models and molecular modeling methods. MoMA-LigPath is available at http://moma.laas.fr. The web server is free and open to all users, with no login requirement.

## INTRODUCTION

In the past, experimental and computational approaches aimed at investigating protein–ligand interactions have mostly focused on the molecular complex, when the ligand is docked in the active site of the protein. However, an increasing amount of research shows that important interactions that may determine protein–ligand specificity and activity occur far away from the active site, during ligand binding or release (1–4). Unfortunately, obtaining accurate experimental data about protein–ligand interactions taking place far from the active site remains difficult. Computational methods may help to better understand such interactions. However, simulating ligand (un)binding, particularly when the active site is deeply buried into the protein, is also a challenging problem for current computational approaches. Some variants of molecular simulation methods have been devised specifically for that. In particular, Steered Molecular Dynamics (SMD) (5) and Random Acceleration Molecular Dynamics (RAMD) (6) have become popular techniques for the simulation of ligand (un)binding. Both methods are based on the same principle: the application of an artificial force to accelerate the ligand diffusion inside the protein. The direction of this force is defined by the user, in the case of SMD, or chosen randomly, in the case of RAMD. Although the interest and the relevance of these methods are not questioned here, it should be noted that this artificial force must be carefully parameterized to avoid strongly biased (inaccurate) results. Monte-Carlo–based techniques have also been proposed to study ligand (un)binding and diffusion (7). They perform a more computationally-efficient exploration of the conformational space than techniques based on molecular dynamics simulations, and do not require additional artificial forces in the molecular force field to accelerate simulations. Nevertheless, all the aforementioned methods remain computationally expensive.

Our group has developed an original approach to simulate protein–ligand (un)binding and other types of large-amplitude (long time-scale) molecular motions, at a low computational cost (8–10). It is based on a mechanistic representation of molecules, and on the use of methods inspired by robot motion planning algorithms to explore their conformational space. We have validated this approach on experimental data, and compared it with other computational methods. We have also successfully applied it to rational enzyme engineering (2,11). Based on this novel methodology, we are developing a computer software called MoMA (for Molecular Motion Algorithms), which implements a collection of robotics-inspired algorithms for the simulation of molecular motions.

MoMA-LigPath is an application built on the MoMA software. Starting from the model of a protein–ligand complex, MoMA-LigPath computes the ligand unbinding path from the active site to the surface of the protein. In the current version, flexibility is considered only for the ligand and the protein side-chains, and only geometric constraints are involved. Computing times for simulating ligand unbinding in such a simplified case usually range from some seconds to a few minutes. Thus, this simple version is particularly well suited for a web server. Even though they satisfy only geometric feasibility, the paths generated by MoMA-LigPath can provide interesting information to biologists and chemists. They can also serve as a first approximation that can be further refined using standard molecular modeling techniques. Note that more sophisticated algorithmic variants implemented in MoMA can consider protein backbone flexibility and energy models (10,12), at the expense of additional computational costs. These variants could be introduced in subsequent versions of the web server or distributed as binaries.

The next section gives explanations on the molecular models and the algorithm used in MoMA-LigPath. It also provides guidelines for users of the web server. After this, some results illustrating the capabilities of the method are presented.

## MATERIALS AND METHODS

### Underlying methods

In recent years, algorithms originally developed for robot motion planning have been extended and applied to solve different problems in computational structural biology (13,14). MoMA-LigPath applies one of these methods, based on the Manhattan-like RRT (ML-RRT) algorithm (15), originally proposed for disassembly path planning of complex objects with articulated parts. ML-RRT is an extension of the Rapidly-exploring Random Tree (RRT) algorithm (16), which iteratively constructs a tree that tends to rapidly expand on the search space with the aim of finding a feasible path between two given states. The main idea of ML-RRT is to divide variables (i.e. conformational parameters) into two groups, called active and passive variables, and to generate their motions in a decoupled manner. Active variables correspond to parts whose motions are essential for the disassembly task, whereas passive variables correspond to parts that should move only if they hinder the motions of other mobile parts (active or passive). The ML-RRT algorithm presents two main advantages compared with the basic RRT. First, ML-RRT can solve problems practically intractable with RRT. Second, it allows to automatically identify which parts should move to find a solution to the disassembly problem.

The version of the ML-RRT algorithm currently implemented in MoMA-LigPath considers the ligand as an articulated mechanism to be disassembled from the protein. All the protein side-chains are also articulated with freely rotatable bonds. For the conformational exploration, the active variables are the parameters defining the pose (position and orientation) of a reference frame associated with the geometric center of the ligand, as well as the ligand bond torsions; the passive variables are the bond torsions of the protein side-chains. Collision avoidance between non-bonded atoms is the only feasibility condition considered for molecular motions. Further explanations about the approach are provided in previous publications (8,10).

### Description of the web server

#### Overview of the Web site

The MoMA-LigPath Web site (http://moma.laas.fr) is structured into a set of pages. A ‘Home’ page gives an overview of the Web site. ‘Demo’ and ‘Help’ pages provide guidance to users. A ‘Contact us’ page is also included for particular requests. The main page, giving access to the application, is the ‘Start a new job’ page. The ‘My jobs’ page, accessible only to registered users, contains a repository of previously submitted jobs. Finally, the ‘References’ page lists some publications related to MoMA-LigPath. We kindly ask users to cite (at least) one of these publications if they use results provided by MoMA-LigPath in their work.

#### Users and usage modes

MoMA-LigPath is free and open to all users. Access as an anonymous user is possible. Nevertheless, we recommend frequent users to create an account. This will enable them to access a record of the jobs they submit to the server, and to be informed about results by email. Some privacy measures are enforced: the data and results of each user are not accessible to others. However, a completely secure communication pipe between each user and the server cannot be absolutely guaranteed.

Two usage modes are available to run jobs on the web server. The ‘simple’ mode is the standard way of using MoMA-LigPath, and should be sufficient in most cases. The ‘advanced’ mode enables the user to locally modify the flexibility of the molecules, and to access some parameters of the ML-RRT algorithm. Further explanations on these two usage modes are provided below.

#### Input files and parameter settings

The main input of MoMA-LigPath is a ‘.pdb’ file containing the atomic coordinates of the protein–ligand complex. It can contain more than one protein and one ligand, as well as other molecules, such as structural waters or ions. However, only one ligand is considered to be mobile in the current version.

When using the ‘advanced’ mode, the user can locally modify the flexibility of molecules by uploading an additional input file with extension ‘.amc’, which is an application-specific file format. Given a ‘.pdb’ file, a template ‘.amc’ file can be generated via the web server, and subsequently modified with any text editor. The ‘.amc’ file format is extremely simple. It contains blocks describing the flexibility of each molecule. For a protein, the block contains one line per residue. Each line contains the residue type, the residue identifier and two binary values defining the backbone and side-chain flexibility, respectively. A value of 0 means that all the dihedral angles of the backbone or of the side-chain are fixed for that residue. A value of 1 means that they can freely rotate. Note that, in the current version of MoMA-LigPath, only the side-chains (and not the backbone) can be defined as flexible. By default, the flexibility of all side-chains is set to 1. Users can block side-chains by changing the values in their corresponding lines to 0. For a ligand, the block contains one line per rotatable bond. Each line contains the identifiers of the four atoms defining the dihedral angle, as well as the upper/lower bounds for the angle value. The user can remove dihedral angles or modify the bounds, which are set to 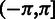 by default. Additional information about the ‘.amc’ file format is provided in the template file header itself.

In both the ‘simple’ and ‘advanced’ modes, the user can optionally tune two basic parameters:

- Percentage of van der Waals radii: This parameter is used for collision detection between non-bonded atoms. Seventy-five percent is a reasonable default value, often used to check atom overlaps in other computational methods. A lower percentage may be necessary to find solutions to constrained problems, which would require some flexibility of the protein backbone. In easy cases, this value can be increased to force the ligand to move along the medial axis of the exit channel.
- Number of paths: Up to 20 solutions can be requested for each job. Some variability can be observed between these solutions because of the random nature of the search performed by ML-RRT. Each solution path will be displayed individually in the results.

Additional parameters are accessible in the ‘advanced’ mode:

- RRT expansion strategy: By default, MoMA-LigPath applies an RRT-Connect strategy to expand nodes during the construction of the search tree. This can be changed to a more basic RRT-Extend strategy, which generally produces shorter local moves, and is thus more computationally expensive. Nevertheless, the RRT-Extend strategy can be more efficient when solving constrained problems. Please refer to the basic literature on the RRT algorithm (16) for further explanations on these strategies.
- Exit distance: The length of the paths to be computed by MoMA-LigPath can be specified by defining the distance (in Å) that has to be reached between the geometric centers of the ligand and the protein. When left unspecified, this distance is automatically computed by MoMA-LigPath, based on the molecule sizes.
- N fail max: This parameter determines the number of consecutive expansion failures after which a node in the search tree is considered ‘exhausted’, and is no longer selected for expansion during the ML-RRT construction. This heuristic is further explained in basic articles on ML-RRT (15). The default value, 50, provides good results in general. This value can be increased for constrained problems.

#### Output and presentation of the results

Registered users receive a notification email when a job terminates on the web server, and results appear on the ‘My jobs’ page. For anonymous users, results are presented on a page having a unique URL.

The main output of MoMA-LigPath is a set of solution paths. Each solution, contained in a separate downloadable archive, is a sequence of ‘.pdb’ files corresponding to intermediate conformations of the molecules along the ligand exit path. The path is discretized in such a way that the maximum displacement of an atom of the ligand between two consecutive frames is ∼0.5 Å. In addition, solution paths can be directly visualized on the results webpage by means of a Jmol applet (17).

For each solution, a file of ‘contacts’ between the ligand and the protein is also generated. This is a text file containing a line for each pair of atoms in contact. A contact is detected if two atoms overlap when their size is increased by 20% with respect to the size used for collision detection during the conformational exploration (e.g. if the path is computed considering 75% of van der Waals radii, contacts are identified for atom overlaps at 95% of van der Waals radii). When the same contact appears for several conformations along the unbinding path, it is listed only for the first conformation. The number of the frame corresponding to this conformation in the sequence of ‘.pdb’ files appears at the beginning of each line in the output file. Information on protein–ligand contacts may be interesting to identify important interactions. For instance, such information can help decision making for protein engineering (2,11).

In addition, an execution report provides information about possible errors in the input file and operations performed by the program. The information contained in this file is important to diagnose MoMA-LigPath failures. A frequent reason for failure is the presence of atom overlaps in the input ‘.pdb’ file. In a preprocessing stage, MoMA-LigPath tries to remove such collisions by slightly perturbing the conformation of the involved side-chains. If not all collisions are removed after a given number of iterations, the program execution stops. In this case, the user may solve the problem on her/his own (e.g. by energy minimizing the model of the complex), or resubmit the job with a reduced percentage of van der Waals radii. MoMA-LigPath may also fail to solve geometrically constrained problems, which would require some flexibility of the protein backbone.

## RESULTS

This section briefly presents results obtained with MoMA-LigPath for the hexameric insulin–phenol complex, which is an interesting test system because of the likely existence of multiple pathways for phenol unbinding. The presented results only aim to illustrate the capabilities of the method, a further biological interpretation being out of the scope of this article. More detailed explanations on the application of robotics-inspired algorithms to simulate protein–ligand unbinding in the context of enzyme engineering can be found in previous publications (2,11).

**Figure 1.**
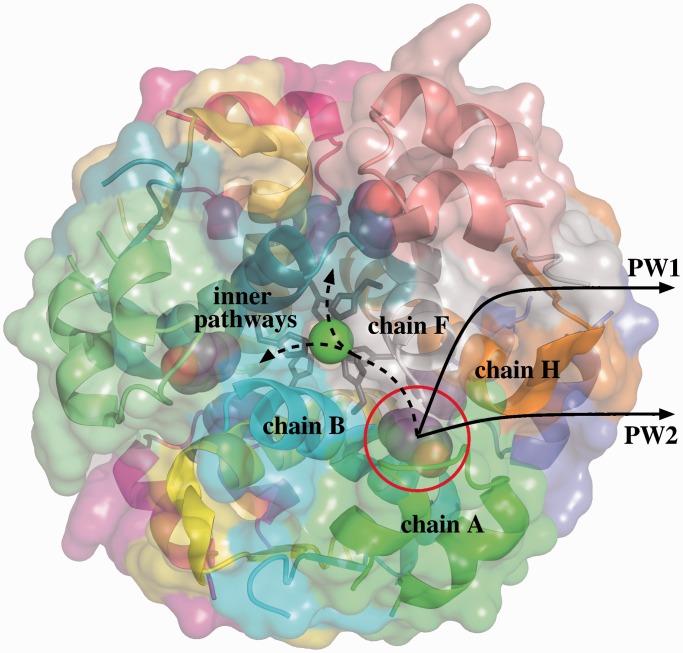
Structure of the R_6_ hexameric insulin–phenol complex. The phenol molecule in the pocket between chains A, B, F and H can follow different unbinding pathways. The two most likely pathways are located at the interface of chains A, F and H. However, diffusion through the inner part of the hexamer is also geometrically feasible. Images of molecular models in this article have been generated using PyMOL (19).

Insulin, in its monomeric active form, is composed of two short peptide chains. In the presence of zinc ions, insulin monomers tend to associate, forming more stable hexameric structures (18). Different conformational states of the insulin hexamer have been observed experimentally. Here, we consider the so-called R_6_ state, which has a 3-fold symmetric toroidal shape (see Figure 1). This conformational state of the insulin hexamer is stabilized by bound phenolic molecules (20). Understanding the mechanism of phenol unbinding is important because it is possibly involved in the conversion of the hexamer into the monomeric active form of insulin (21). Note that the study of the hexameric insulin–phenol complex and of the hexamer–monomer conversion are of interest in pharmacology for the treatment of type 1 diabetes.

The structure of the R_6_ insulin hexamer, determined by radiographic crystallography, is available in the Protein Data Bank (22), with PDB ID: 1ZNJ. The corresponding ‘.pdb’ file was used as input for MoMA-LigPath. The R_6_ insulin hexamer presents six hydrophobic pockets containing bound phenol molecules. MoMA-LigPath was applied to simulate the unbinding of one of them: the one located in the binding pocket between chains A, B, F and H. The other phenol molecules were kept in the input file, and considered to be static molecules. The side-chains of the six histidines interacting with the zinc ions where blocked by editing the input ‘.amc’ file as explained in the previous section. The percentage of van der Waals radii used for collision detection between non-bonded atoms during the conformational exploration was set to 75 (i.e. the default value). Hydrogen atoms were not added. This is acceptable because the solutions provided by MoMA-LigPath are not expected to be an accurate representation of unbinding paths, but simply a first approximation.

**Figure 2.**
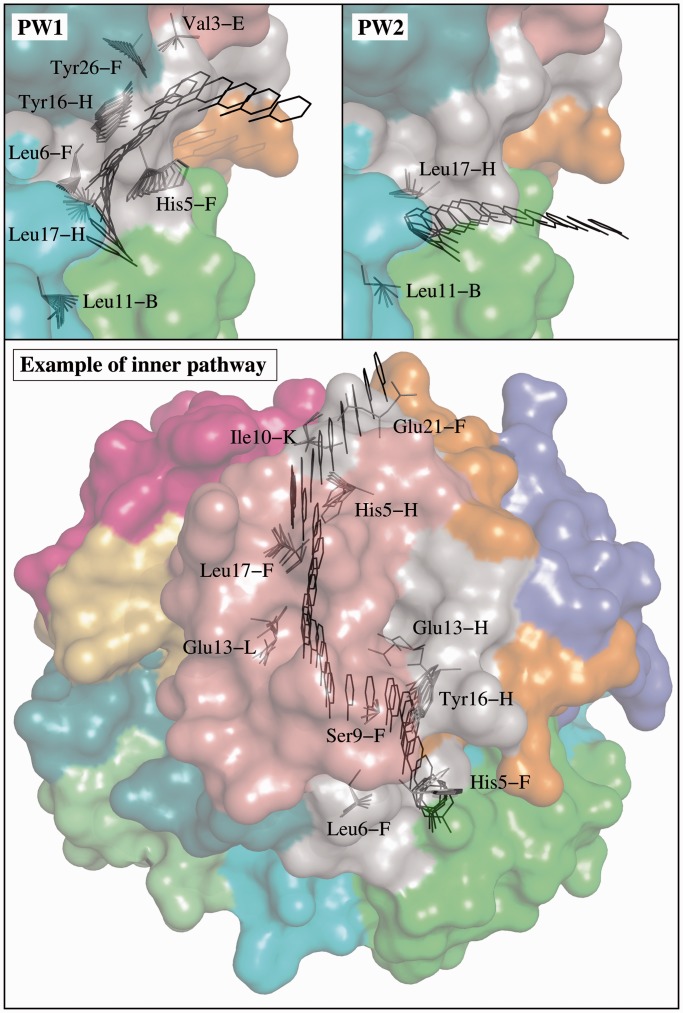
Different paths for phenol unbinding from the R_6_ insulin hexamer obtained by MoMA-LigPath. The location of the phenol molecule and the conformations of moving side-chains are represented for some intermediate frames. The two images at the top correspond to paths following the most likely unbinding pathways: PW1 and PW2. The image at the bottom illustrates one of the pathways going through the inner part of the insulin hexamer.

MoMA-LigPath was run to simulate 20 phenol unbinding paths. To emphasize the computational efficiency of the method, we would like to mention that the average computing time for one solution was <10 s on a single processor. A significant variability in the solutions can be observed. In most simulations, the phenol molecule exits following pathways at the interface between chains A, F and H (see Figure 1). Such paths can be clustered into two groups, which we refer to as pathway 1 (PW1) and pathway 2 (PW2), following the notation used in related work (21). Twenty-five percent of the paths (5 over 20) follow PW1. The ligand finds a passage between residues Ile10-A and His5-F, by inducing a significant conformational change of His5-F. Note that ring-flipping of His5 has been suggested by other computational and experimental studies on this system (21). The ligand also induces the motion of the side-chain of Tyr16-H, which itself induces the motion of Tyr26-F. The conformations of other residue side-chains are also slightly perturbed by phenol unbinding following PW1. PW2 is the most frequent pathway observed in the solutions provided by MoMA-LigPath: 60% of the paths follow PW2. After a quick analysis of the solutions using a molecular viewer, one can observe that PW2 is the shortest and geometrically easiest pathway between the binding pocket and the protein surface, which explains the highest probability to obtain this type of solution. The ligand follows a narrow, partially open channel, and induces slight conformational changes of only a few residues, mainly of Leu17-H. Remarkably, a combination of RAMD and SMD methods also pointed out PW2 as the most probable pathway for phenol unbinding (21). Figure 2 shows some intermediate frames of two solutions: one following PW1 and the other following PW2. The ligand and moving side-chains are represented. The figure also illustrates another type of pathway that was not reported in related work (21). Surprisingly, in a few simulations (3 over 20), the ligand diffuses inside the insulin hexamer before finding an exit pathway. Indeed, the phenol molecule moves at the interface of insulin monomers toward the center of the hexamer, as indicated by the dashed line in Figure 1, eventually finding exit channels that are the symmetric counterparts of PW1 and PW2. Note that the other phenol molecules, which are considered to be static in the simulations, do not obstruct these pathways. In two of the simulations, the ligand exits through a pathway similar to PW2 between chains F, H and K. One of such inner pathways is represented in Figure 2. In another simulation, the ligand follows a pathway similar to PW1 between chains C, J and L. Exit through all the other pathways symmetric to PW1 and PW2 seems to be geometrically feasible. Note, however, that none of the 20 runs of MoMA-LigPath reported here, neither of the 100 additional runs performed in the same conditions, was able to find a third class of pathway (PW3) obtained by RAMD simulations as reported in (21). PW3 is located at the interface between the two chains of the insulin monomer. It is a narrow corridor, involving steric interactions of phenol with many residues. The fact that PW3 was not found by MoMA-LigPath means that it is geometrically unlikely, or even impossible, if the protein backbone does not deform. MoMA-LigPath was run again 20 times with a reduced atom size, namely 60% of van der Waals radii, to simplistically emulate slight fluctuations of the backbone. In this case, 10% of the solutions followed PW3. Nevertheless, as some of the results obtained with such a reduced atom size can be unrealistic, we prefer not to argue about the existence of this pathway type. More generally, a further analysis of the results presented in this section, considering energies, would be necessary to yield a more accurate model of phenol unbinding from the R_6_ insulin hexamer.

## CONCLUSION

This article has presented the MoMA-LigPath web server, which, to the best of our knowledge, is the first web server for the simulation of protein–ligand unbinding. MoMA-LigPath is based on a mechanistic representation of the molecular system, considering partial flexibility, and on the application of a robotics-inspired algorithm to explore the conformational space. The simplicity of the molecular model, together with the efficiency of the exploration algorithm, allow for the simulation of protein–ligand unbinding within short CPU time, which enables an implementation as a web application. Results of a battery of tests on MoMA-LigPath show that CPU times generally range from some seconds to a few minutes. The performance of the method depends on the geometric difficulty, which is mainly related to the narrowness/topology of the access channel, rather than to the size of the molecules.

Our aim with this web server is to provide easy accessibility to the methods we develop, which may interest a large scientific community. In particular, MoMA-LigPath can be an interesting tool for protein engineering, to help decision making for site-directed mutagenesis experiments. More generally, information on local protein–ligand interactions taking place far away from the active site may help understanding the overall molecular interaction mechanism. Nevertheless, we would like to mention that, although geometry can play a determinant role, it is well known that electrostatics, pH and other conditions that are not considered in this work can also dramatically affect protein–ligand (un)binding. Therefore, solutions provided by MoMA-LigPath have to be considered as a first approximation that may require subsequent refinement and analysis using more accurate models. In conclusion, MoMA-LigPath is not proposed as an alternative method, but rather as a complementary tool to be used in tandem with other computational and experimental methods.

## FUNDING

French National Research Agency (ANR) under projects GlucoDesign and ProtiCAD [project number ANR-12-MONU-0015-01] (in part). Funding for open access charge: ANR under project ProtiCAD.

*Conflict of interest statement*. None declared.
